# Pulmonary ^18^F-FDG uptake helps refine current risk stratification in idiopathic pulmonary fibrosis (IPF)

**DOI:** 10.1007/s00259-017-3917-8

**Published:** 2018-01-16

**Authors:** Thida Win, Nicholas J. Screaton, Joanna C. Porter, Balaji Ganeshan, Toby M. Maher, Francesco Fraioli, Raymondo Endozo, Robert I. Shortman, Lynn Hurrell, Beverley F. Holman, Kris Thielemans, Alaleh Rashidnasab, Brian F. Hutton, Pauline T. Lukey, Aiden Flynn, Peter J. Ell, Ashley M. Groves

**Affiliations:** 1 Respiratory Medicine, Lister Hospital, Stevenage, UK; 2 Radiology Department, Papworth Hospital, Papworth Everard, Cambridge, UK; 3 CITR, UCL and Interstitial Lung Disease Centre, UCLH, London, UK; 4 Institute of Nuclear Medicine, University College London/Hospital, 5th Floor, 235 Euston Road, London, NW1 2BU UK; 5 Interstitial Lung Disease Unit, Royal Brompton Hospital, London, UK; 6 Fibrosis and Lung Injury DPU, GlaxoSmithKline R&D, Stevenage, UK; 7 Statistical Consultancy, Exploristics, Belfast, UK

**Keywords:** Interstitial lung disease, Positron-emission tomography and computed tomography, Fluorine-18 FDG

## Abstract

**Purpose:**

There is a lack of prognostic biomarkers in idiopathic pulmonary fibrosis (IPF) patients. The objective of this study is to investigate the potential of ^18^F-FDG-PET/ CT to predict mortality in IPF.

**Methods:**

A total of 113 IPF patients (93 males, 20 females, mean age ± SD: 70 ± 9 years) were prospectively recruited for ^18^F-FDG-PET/CT. The overall maximum pulmonary uptake of ^18^F-FDG (SUV_max_), the minimum pulmonary uptake or background lung activity (SUV_min_), and target-to-background (SUV_max_/ SUV_min_) ratio (TBR) were quantified using routine region-of-interest analysis. Kaplan–Meier analysis was used to identify associations of PET measurements with mortality. We also compared PET associations with IPF mortality with the established GAP (gender age and physiology) scoring system. Cox analysis assessed the independence of the significant PET measurement(s) from GAP score. We investigated synergisms between pulmonary ^18^F-FDG-PET measurements and GAP score for risk stratification in IPF patients.

**Results:**

During a mean follow-up of 29 months, there were 54 deaths. The mean TBR ± SD was 5.6 ± 2.7. Mortality was associated with high pulmonary TBR (*p* = 0.009), low forced vital capacity (FVC; *p* = 0.001), low transfer factor (TLCO; *p* < 0.001), high GAP index (*p* = 0.003), and high GAP stage (*p* = 0.003). Stepwise forward-Wald–Cox analysis revealed that the pulmonary TBR was independent of GAP classification (*p* = 0.010). The median survival in IPF patients with a TBR < 4.9 was 71 months, whilst in those with TBR > 4.9 was 24 months. Combining PET data with GAP data (“PET modified GAP score”) refined the ability to predict mortality.

**Conclusions:**

A high pulmonary TBR is independently associated with increased risk of mortality in IPF patients.

**Electronic supplementary material:**

The online version of this article (10.1007/s00259-017-3917-8) contains supplementary material, which is available to authorized users.

## Introduction

Interstitial lung disease (ILD) has an incidence of ~57/100,000 per year and is associated with significant morbidity [1]. The ILDs consist of a heterogeneous group of diseases with varying amounts of interstitial inflammation and fibrosis [2]. However, there is heterogeneity in outcome, with survival in idiopathic pulmonary fibrosis (IPF) particularly poor. Some patients gradually deteriorate, some undergo stepwise progression, whilst others decline rapidly. Moreover, much of the prognostic data heralds from an era when the criteria for diagnosing IPF were less well and differently defined than at present [2–4].

Positron emission tomography (PET) offers the ability to non-invasively investigate cellular metabolism in vivo. PET studies in animals have yielded valuable insights into the biology of IPF and ILD [5, 6], and there is encouraging evidence that PET may aid the development of therapeutic interventions to treat these debilitating conditions [7]. It has recently been demonstrated that^18^F-fluorodeoxyglucose (^18^F-FDG) PET signal is consistently raised and can be objectively measured in patients with IPF [8]. Moreover, these PET signals are shown to be stable and reproducible [9].

The prevailing theory of IPF is that alveolar epithelial damage results in a chronic wound-healing response that leads to self-propagating scar formation and end-stage fibrosis [10]. ^18^F-FDG uptake by tissues is a marker of glucose utilization, correlating with tissue metabolism. Our hypothesis was that although the cellular basis of the FDG-PET signal is unclear, on-going metabolic activity, in areas of scarred lung, would indicate a process that could be manipulated therapeutically. In addition, a significant correlation between FDG-PET signal and disease progression would strengthen the validity and usefulness of this signal as a novel biomarker in IPF [11].

With the use of current treatment (e.g., anti-fibrinolytic therapy) in IPF patients [12, 13] and given the rationale for recommending these drugs as set out in some guidelines [14, 15], it has been recently highlighted that there is an urgent need for biomarkers and end points in order to develop therapy and treatment regimens for patients with IPF [3].

In this study, we investigate the potential for baseline measures of pulmonary ^18^F-FDG PET signal to predict survival in patients with IPF compared to the more established GAP (gender age and physiology) prognostic score [16].

## Methods

### Patients

From 2008 to 2017, there were a total of 113 (93 male, 20 female, mean age 70 ± 9 years) prospectively and consecutively consented patients with IPF who underwent ^18^F-FDG PET/CT from a single institution. All patients underwent full clinical assessment including multidisciplinary team (MDT) review, baseline pulmonary function tests (PFTs), and high-resolution computed tomography (HRCT) evaluation. Infection and neoplasia were excluded on clinical and radiological grounds. The diagnosis of IPF was made on clinico-radiological grounds following MDT review. The study had ethics board approval and all patients gave written informed consent.

### Patient follow-up

The patient follow-up period was defined from date of scan to death (all causes, as previously adopted in prognostic studies [16]) or 9 years. Patient survival was confirmed by the use of patient charts, electronic database, primary health care physician records, or telephone interview.

### PET/CT acquisition

PET/CT imaging was performed after the diagnosis was made following MDT review described above and all images were acquired on the same PET CT instrument (VCT PET/64-detector CT instrument, GE Healthcare Technology, Waukesha, WI, USA). Three imaging sequences of the thorax were performed whilst the patient remained supine on the table throughout. A CT was performed for attenuation correction. Maintaining the patient position, a whole-body ^18^F-FDG PET emission scan (8 min per bed position) was performed 1 h after injecting 200 MBq of ^18^F-FDG and covered an area identical to that covered by CT. Next, maintaining the patient position, a deep inspiratory breath-hold diagnostic high-resolution CT (HRCT) was performed using the following parameters: 64 × 1.25-mm detectors, a pitch of 0.53, and 1.25-mm collimation (120 kVp and 100 mAs).

### Image analysis

#### Observers

PET images were analyzed by a PET radiologist and senior PET technologist with > 5 years’ experience in quantifying pulmonary ^18^F-FDG PET uptake in IPF. CT images were reviewed by a dedicated thoracic radiologist, independent of the PET CT analysis.

#### Image display and processing

**Fig. 1:**
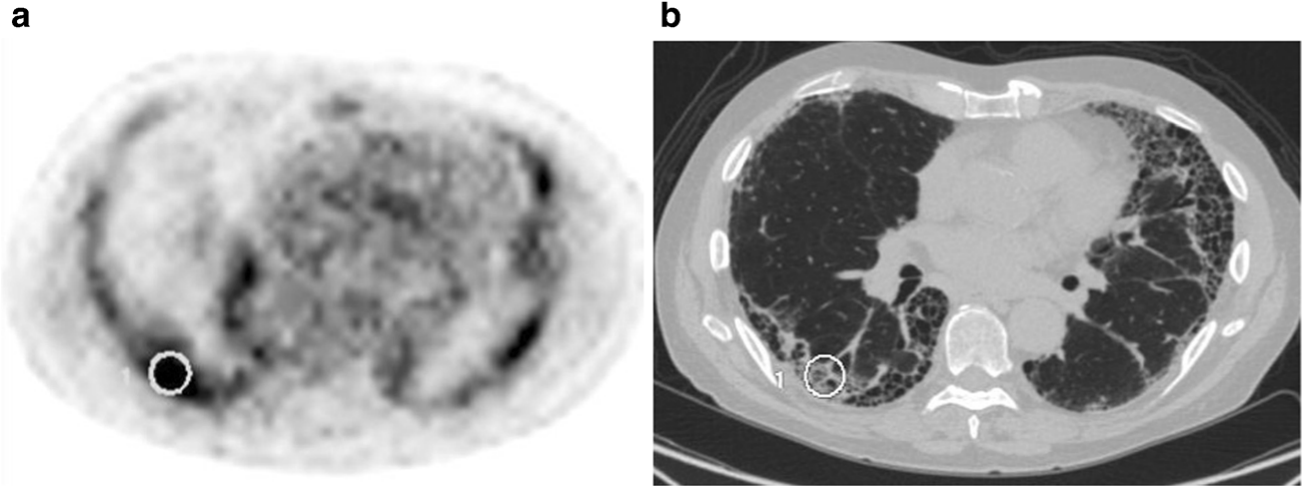
Co-registered PET (**a**) and CT (**b**) of a patient with IPF showing region of interest placement as part of measuring maximal pulmonary ^18^F-FDG uptake [7]. The *dark grey/black* regions are regions of high^18^F-FDG metabolism on the PET image (**a**)

All images were loaded onto an ADW (GE Healthcare Technology, Waukesha, WI, USA) workstation. All datasets underwent image processing as previously described in detail [7]. Using a region of interest, the area of most intense pulmonary ^18^F-FDG uptake was identified and the highest image value (SUV_max_) measured (see Fig. 1).

In addition, the region of pulmonary parenchyma with the lowest SUV was identified as previously described and was shown to have high inter- and intra-observer reproducibility [7]. In all cases, this region was confirmed by the dedicated thoracic radiologist to conform to morphologically normal lung parenchyma on co-registered CT. The lowest image value (i.e., SUV_min_) in this region was recorded as a measure of the background lung uptake and in turn to calculate the lung target-to-background ratio (TBR = SUV_max_/SUV_min_) [7]. TBR can be interpreted as a measure of variation of FDG uptake in the lung where a TBR close to 1 indicates relatively uniform uptake. In addition, tissue-to-blood ratios have been reported in several studies to be highly correlated with Ki [17–19], therefore we also measured lung-to-blood background levels by drawing an ROI as described by the European Association of Nuclear Medicine position paper [20], and thus “TBR blood” was calculated as lung SUV_max_ divided by average value of the blood SUV_mean_.

#### GAP calculation

GAP calculation based on the four variables (gender (G), age (A), and 2 lung physiology variables (P) forced vital capacity (FVC) and transfer factor (TLCO) as previously described was computed [16]. This comprised a model using continuous predictors (GAP calculator) and a simple point-scoring system (GAP index), which varies from 0, potentially indicating a good outcome, to 8, potentially indicating a worse outcome.

Based on the GAP index, the three stages (stages I, II, III) identified are: GAP stage I included GAP index 0, 1, 2, 3; GAP stage II included GAP index 4, 5; and GAP stage III included GAP index 6, 7, 8.

### Statistical analysis

Statistical analyses were performed using SPSS for Windows version 19.0 (IBM, Armonk, NY, USA).

#### Univariate survival analysis

The relationships of SUV_max_, SUV_min_, TBR, and TBR blood with patient survival were assessed using univariate Kaplan–Meier (KM) survival analysis. Median values for these parameters were used as thresholds (cut-offs) to separate the survival plots (poor and good prognostic groups) and the difference in the survival plots was further evaluated using non-parametric log-rank test. KM curves for patients above and below the median cut-off for each PET parameter were constructed to display the proportion of patients surviving at a given time.

#### Cross-validation

Although the use of median value as a cut-off is unbiased, to further reduce bias, cross-validation was undertaken for each of the significant univariate PET markers of survival within the respective patient sub-group [21]. Because of the sample size, cross-validation could not be carried out on the basis of a sample split (training and validation). Instead, the statistically recognized k-fold (e.g., k = 4 in our study) cross-validation procedure was carried out [21]. See Supplemental Material for further detail.

#### Multivariate Cox regression analysis

Multivariate step-wise forward Wald–Cox regression was used to determine which significant cross-validated PET parameters (SUV_max_, SUV_min_, TBR, and TBR blood) were independent of the GAP (GAP index and GAP stage) parameters in predicting patient survival.

#### Modeling PET data with GAP analysis

The GAP scores were reclassified and modified based on the PET parameters to ascertain if the combinations were synergistic and improved the ability to predict survival.

For the PET-modified GAP calculation, we proposed adding a fourth PET variable (SUV_max_ or SUV_min_ or TBR or TBR blood that demonstrated the best prognostic ability in our study, based on the above univariate Kaplan–Meier survival analysis, cross-validation, and multivariate Cox regression analysis) to the existing GAP index calculation. For each patient, the best PET marker was binarized, based on the median cut-off, as an adverse (coded as 1) or favorable (coded as 0) PET signal similar to the coding employed in GAP calculation. This was further added to the existing GAP index calculation where the modified GAP index ranged from 0 to 9. For example, if a patient with original GAP index “0” had an adverse PET marker i.e., “1”, the modified GAP index would be “0 + 1” = “1”. Conversely, if the patient with original GAP index “0” had a favorable PET marker i.e., “0”, the modified GAP index would be “0 + 0” = “0”. So the “new” modified GAP index (mGAP) ranged from 0 to 9 in comparison to the original GAP index, which ranged from 0 to 8. Based on the mGAP index, we redefined the mGAP stages (stages I, II, III) as follows: mGAP stage I includes mGAP index 0, 1, 2, 3; mGAP stage II includes mGAP index 4, 5, 6; and mGAP stage III includes mGAP index 7, 8, 9.

A second PET altered (risk stratified) GAP model was also formulated where GAP stage was risk stratified (S-GAP) is presented in the Supplemental Section.

#### C-index (goodness of fit)

To further assess how good the modified GAP stage was at predicting a binary outcome (mortality) in comparison to GAP stage, the concordance statistic (C-index or C-statistic) was measured as the area under the receiver operating characteristic (ROC) curve along with the 95% confidence interval. In all analyses, *p* values < 0.05 were considered significant.

## Results

**Fig. 2:**
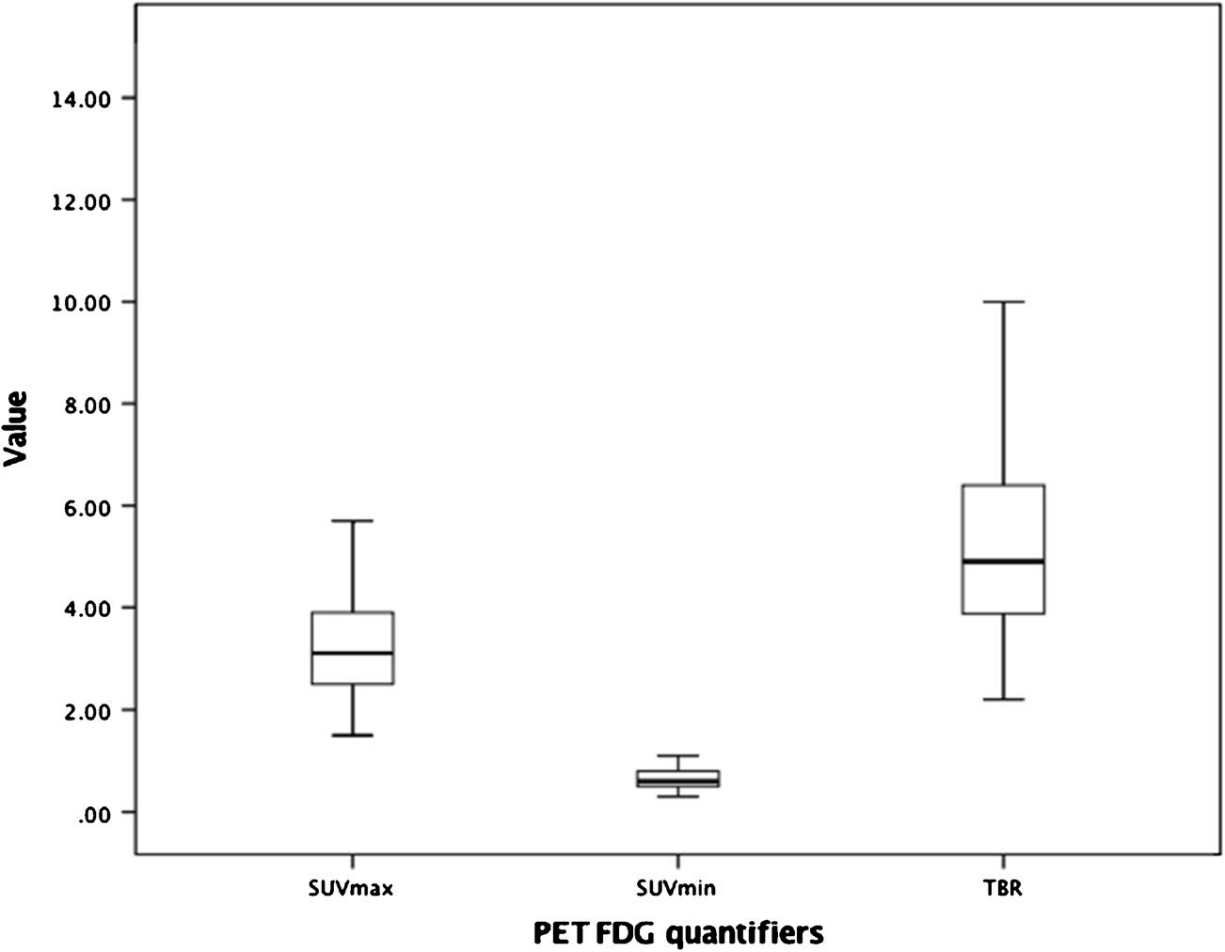
Box plots highlights the distribution of the individual PET markers (SUV_max_, SUV_min_, TBR) for the entire patient population

There were 113 IPF patients in total. Eight percent of patients were known to have a co-morbidity of cancer, 18% cardiovascular disease 6% diabetes and 41% other diseases. Twenty patients were known to have a history of anti-fibrotics, a further 44% had a trial of steroids, and 17% immunosuppressants, but not at the time of scanning. The mean ± 1 SD follow-up period was 29 ± 25.2 months. There were 54 deaths during the follow-up. The mean ± SD for SUV_max_ was 3.4 ± 1.4, SUV_min_ (background lung activity) was 0.7 ± 0.2 and the mean TBR 5.6 ± 2.7 (Fig. 2).

The mean ± SD for FVC (percentage predicted) was 74.0% ± 18.2. The mean ± SD for TLCO (percentage predicted) was 45.3 ± 14.4. GAP analysis was performed on 112 patients as FVC was unobtainable in one patient. KM analysis showed that patients that demonstrated a high pulmonary FDG uptake value had poorer survival scores (Table 1). Of the 14 patents who were treated with pirfenidone and six who were treated with nintedanib, no statistically significant survival difference was detected in these groups.

**Table 1:** Summary of the cohort associations with survival incorporating the components of GAP analysis. PET (SUV_max_, SUV_min_, TBR and TBR blood), age, gender, FVC, TLCO, and GAP (index and stage) parameters, median cut-off (direction indicates poor prognosis), hazard ratio (HR), 95% confidence interval (CI) and their association with mortality (as assessed by log rank test from Kaplan–Meier analysis). Statistically significant results are shown in bold.

Parameter	IPF		
	Median	HR (95% CI)	*p* value
SUV_max_	> 3.1	1.3 (0.8–2.3)	0.317
SUV_min_	> 0.6	1.2 (0.7–2.3)	0.490
TBR	**> 4.9**	**2.1 (1.2–3.6)**	**0.009**
TBR blood	> 2.1	1.5 (0.9–2.6)	0.147
Age	> 71.0	1.5 (0.9–2.7)	0.138
Gender	Male	1.6 (0.7–3.7)	0.275
FVC	**< 72.5%**	**2.5 (1.4–4.5)**	**0.001**
TLCO	**< 45.0%**	**3.4 (1.8–6.8)**	**<0.001**
GAP index	**> 4**	**2.9 (1.4–5.9)**	**0.003**
GAP stage	**> II**	**2.9 (1.4–5.9)**	**0.003**

**Fig. 3:**
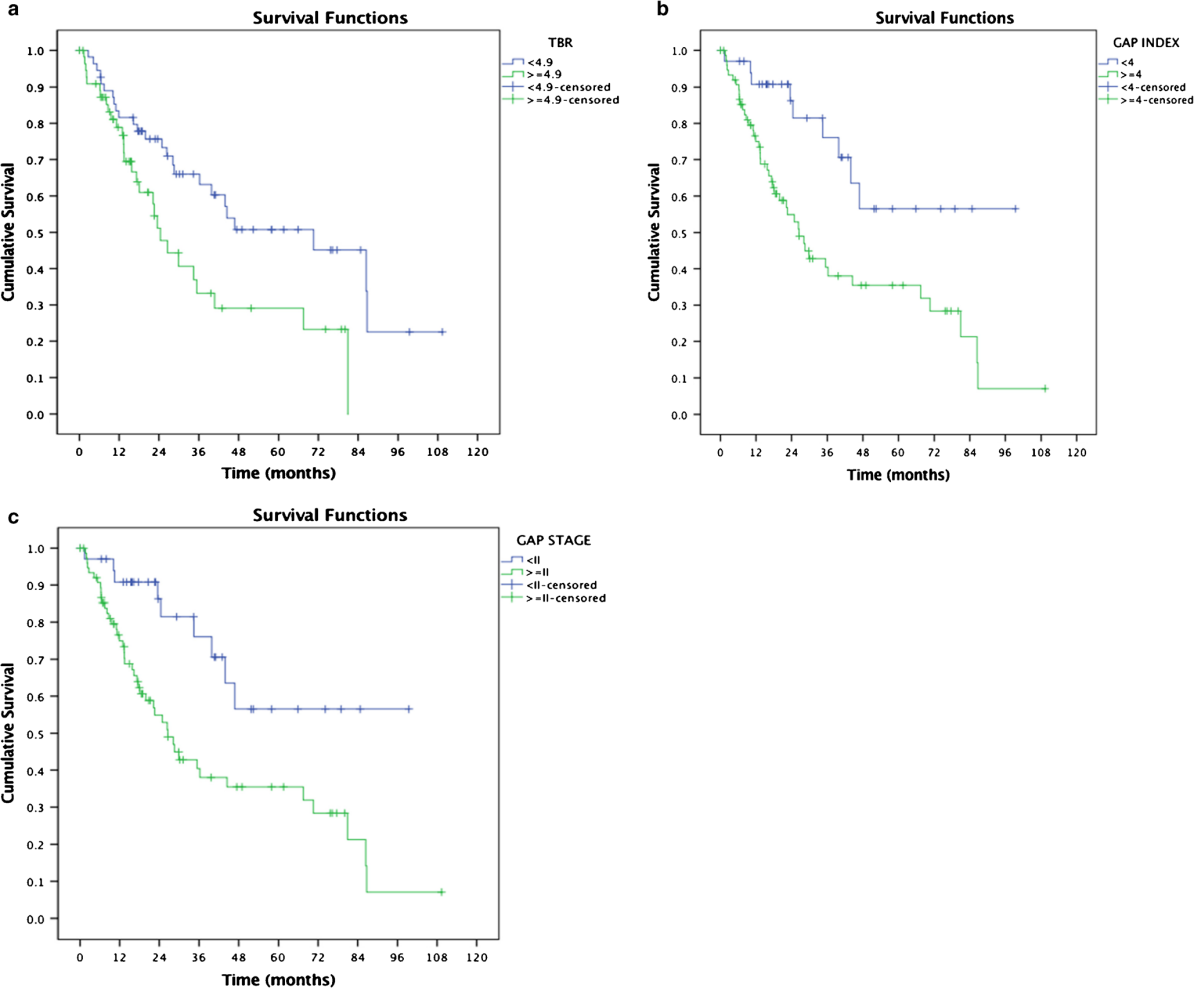
**a** Kaplan–Meier survival curve analysis demonstrating a relationship between TBR (cut-off value of 4.9 = median) and survival in IPF patients after cross-validation. **b** Kaplan–Meier survival curve analysis demonstrating a relationship between GAP index (cut-off value of 4) and survival in IPF patients. **c** Kaplan–Meier survival curve analysis demonstrating a relationship between GAP stage (cut-off value of II) and survival in IPF patients

Of the 113 IPF patients, there was a significant association (Fig. 3a) between uptake of pulmonary ^18^F-FDG and survival (TBR, *p* = 0.009, before cross-validation), where patients below the median cut-off (< 4.9) had a better prognosis than patients with a TBR > 4.9 (hazard ratio: 2.1, 95% CI: 1.2–3.6). After the fourfold cross-validation, the median cut-off for each of the individual (four) folds was found to be the same as the entire population, whereby patients below the median cut-off (< 4.9) had a better prognosis than patients with a TBR > 4.9 had worse prognosis (hazard ratio: 2.1, 95% CI: 1.2–3.6).

Patients with a TBR below a threshold of 4.9 had a 3-year survival of > 65% compared to a 3-year survival of < 35% for patients with a TBR above the threshold. Below the threshold, ~ 75% survived at 2 years and ~ 50% at 5 years, whilst survival was ~ 50 and 30%, respectively, above the threshold. The 50% mortality (median survival) below the threshold was ~ 6 years, whilst above the threshold it was ~ 2 years.

There was a significant association (Fig. 3b) between GAP “index” and survival (GAP index, *p* = 0.003), where patients below the median cut-off (< 4) had good prognosis and patients > 4 had poor prognosis with a hazard ratio of 2.9 (95% CI: 1.4–5.9).

In patients with a GAP index below a threshold of 4, ~ 80% survived at 3 years, and ~ 45% at 3 years above the threshold. In patients with a GAP index below a threshold of 4, ~ 86% survived at 2 years and ~ 56% at 5 years, whilst survival was ~ 55 and 35%, respectively, above the threshold. The 50% mortality below the threshold was greater than 8 years whilst above the threshold it was less than 2.5 years.

There was a significant association (Fig. 3c) between GAP stage and survival (GAP stage, *p* = 0.003), where patients below the median cut-off (< II) had good prognosis and patients > II had poor prognosis with a hazard ratio of 2.9 (95% CI: 1.4–5.9).

In patients with a GAP “stage” below a threshold of II, ~ 75% survived at 3 years whilst survival was ~ 40% above the threshold. In patients with a GAP stage below a threshold of II ~ 86% survived at 2 years and ~ 56% at 5 years, whilst survival was ~ 55 and 35%, respectively, above the threshold. The 50% mortality below the threshold was greater than 8 years whilst above the threshold it was less than 2.5 years.

The effect of the individual components of GAP (FVC and TLCO) on PET survival stratification are shown in the Supplementary Section.

### Cox analysis to assess the independence of PET (TBR) and GAP

PET parameter (TBR) was independent of GAP analysis. By including TBR and GAP index in a Cox regression model, TBR (median threshold > 4.9, HR: 2.092, 95% CI: 1.192–3.674, *p* = 0.010) and GAP Index (median threshold > 4, HR: 2.894, 95% CI: 1.409–5.945, *p* = 0.004) were both independent predictors of survival.

By including TBR and GAP stage in a Cox regression model, TBR (median threshold > 4.9, HR: 2.092, 95% CI: 1.192–3.674, *p* = 0.010) and GAP stage (median threshold: > II, HR: 2.894, 95% CI: 1.409–5.945, *p* = 0.004) were both independent predictors of survival.

### Modeling PET-derived TBR by combining with GAP analysis

**Fig. 4:**
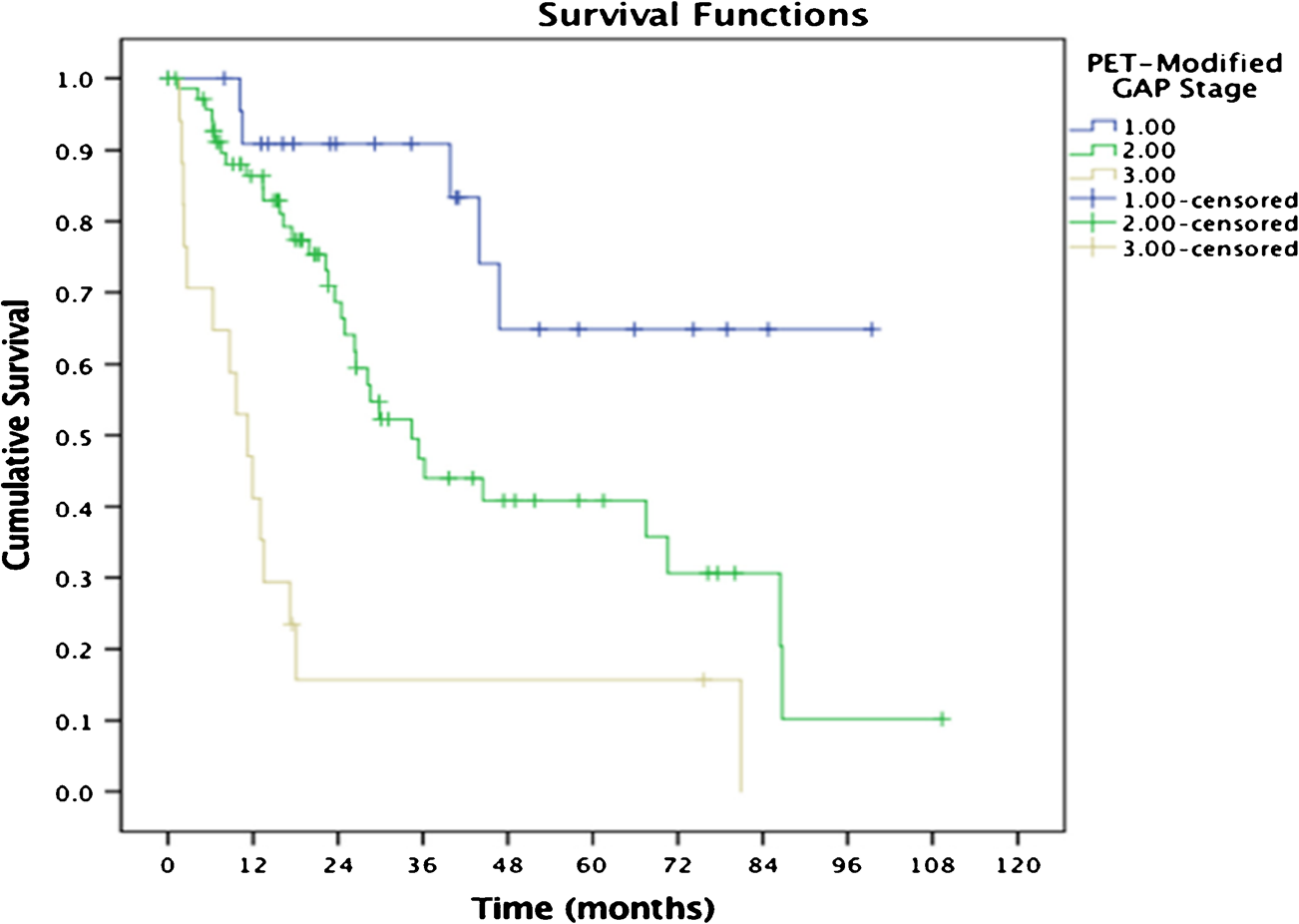
KM-derived survival curves from the modified GAP groups showing that PET can refine the mortality prediction (see also Fig. 3b and c, and Table 3 above). *The difference in the survival curves based on the PET modified GAP stage yielded a *p* < 0.001 (log-rank test)

There was synergy in survival associations (Tables 2 and 3, Fig. 4, and Supplementary Table 1 see below).

**Table 2:** Three-year mortality for the univariate PET, PFT, and GAP indices as stratified based on above/below the threshold identified in Table 1

	3-year mortality
TBR < median, (*n* = 56)	35%
TBR > median, (*n* = 57)	70%
GAP I (*n* = 35)	24%
GAP II (*n* = 50)	56%
GAP III (*n* = 27)	65%
FVC > median (*n* = 56)	29%
FVC < median (*n* = 56)	68%
TLCO > median (*n* = 45)	19%
TLCO < median (*n* = 47)	73%

**Table 3:** Comparison between original GAP 3-year mortality PET versus PET modified GAP Stage (mGAP). The PET modified GAP stage classification shows improved risk stratification compared to GAP on its own especially in the stage I and III groups

	3-year mortality
GAP I (*n* = 35)	24%
mGAP I (*n* = 24)	9%
GAP II (*n* = 50)	56%
mGAP II (*n* = 71)	53%
GAP III (*n* = 27)	65%
mGAP III (*n* = 17)	84%

#### PET-modified GAP calculation

The findings from the modified GAP using the TBR are presented in Table 3. It shows improved risk stratification over the original GAP analysis and this additional benefit is further shown in the ROC analysis in the next section below.

### C-index

Area under the ROC curve (AUC) along with the 95% confidence interval and *p* value for GAP stage and m-GAP stage are as follows: 0.667 (0.567–0.767, *p* = 0.002) and 0.694 (0.598–0.791, *p* < 0.001) demonstrates increased ability of m-GAP to predict patient mortality.

## Discussion

In a population of 113 IPF patients, we have shown that baseline objective measures of ^18^F-FDG uptake on PET are related to patient survival. High pulmonary ^18^F-FDG uptake (TBR) was associated with poor survival and was independent of GAP stage. The GAP classification in our population was also a strong survival predictor, but as is recognized, many patients were unable to perform TLCO and PFTs can be skewed in IPF patients with emphysema. Moreover, the GAP classification measurements were synergistic with the prognostic potential of lung pulmonary ^18^F-FDG uptake. As shown in Table 3 above, PET signal reclassifies a subset of patients at GAP stage 1, as modified GAP stage 2 despite their relatively preserved lung function. Thus, even in groups of patients with good PFTs, the pulmonary ^18^F-FDG uptake could identify sub-populations of patients that would perform poorly. This may have clinical implication as treatment recommendations can depend on PFT performance [14, 15]. As such, pulmonary ^18^F-FDG uptake measured with PET is a potential prognostic biomarker in IPF.

The synergies between PET and GAP (Table 3 and Supplemental Table 1) are interesting. In GAP stage III patients, we identified IPF patients that have a better outcome (similar to GAP II) than the other GAP III patients, whose 3-year mortality was nearly 90%. Likewise, there were patients in GAP stage I that had high ^18^F-FDG uptake, whose outcome was so poor that they may have benefited from treatment. Currently, IPF patients with FVC of 80% predicted and above are considered to have mild disease and treatment with pirfenidone or nintedanib is not recommended in the UK by the National Institute of Clinical Excellence [14, 15]. However, there was evidence of benefit of anti-fibrotics even in patients with mild disease (FVC > 80% predicted) in the original studies [16, 17]. Our data show (see supplement Fig. 1) that a high pulmonary TBR may identify a sub-group of patients that have a poor survival despite only mildly impaired lung function (FVC > 80% predicted); compared to a group of patients with moderately impaired lung function (FVC < 80% predicted) that progress slowly. Thus, the use of ^18^F-FDG PET in this context raises the possibility of selecting patients for pirfenidone or nintedanib in a wider patient population.

Given the heterogeneous outcome of IPF patients, predictors of mortality in these patients are important for many reasons. Patients may need to understand their prognosis to plan for the future and assist them in their decision-making before taking experimental therapeutics. The patients’ physicians need information to determine the required intensity of follow-up, to enable them to balance the risks of entering their patients into drug trials versus the possible benefits, and also guide them in the timing of referral for lung transplant assessment and the listing of patients on an active transplant waiting list. As such, we show that those IPF patients with a pulmonary FDG uptake with a TBR > 4.9 have twice the risk of death compared to those with low measurements (see Cox models in the Results section above).

Given the inherent problems in using one modality for prognosis there has been much interest in clinical models of risk prediction that take into account age, sex, lung function, and other parameters, such as imaging in some cases, to build a risk score. These have been aimed mainly at IPF, although the original GAP (gender, age, physiology) model has been modified for use across all ILDs and shown to perform well [10, 22, 23]. However, even using these models, there currently remains a lack of survival endpoints. This has prompted the drive for serum biomarkers that can prospectively predict patients at most risk of disease progression. For example, in the PROFILE study, concentrations of 11 novel epitopes derived from matrix metalloprotease (MMP)-degraded ECM proteins were related to subsequent progression of IPF [24]. Future studies may be aimed at seeing if combinations of clinical models, serum biomarkers, and functional imaging can further refine the theranostic approach in IPF.

Since the prognosis is generally poor in IPF, effective treatments are required and the development of new therapeutic agents is urgently needed. However, at present, there is a lack of useful biomarkers to detect or monitor disease activity in IPF or predict survival. Using the currently available clinical predictors, such as GAP and survival data, trials of novel agents will be long in duration and require costly clinical studies [3, 4, 25]. Therefore, if the pulmonary uptake of ^18^F-FDG on PET is associated with survival in IPF patients, it would be interesting to investigate whether this could be a potential cost-effective response biomarker for drug development.

HRCT remains the main diagnostic imaging investigation in IPF. However, there are more limited data showing the prognostic use of HRCT. Previous investigations have provided associations between survival data and visual scoring/grading systems of HRCT findings in acute exacerbation, a specific situation in which the overall prognosis is generally dire [26, 27]. In the more general prognostic bio-marker role of baseline HRCT in ILD, one single-center study showed that baseline HRCT could be used to predict survival [28] and a large study of mild-to-moderate IPF showed that the extent of honey combing and reticulation was inversely associated with survival [29]. The latter study was a post hoc analysis to a phase III intervention study and thus potentially complicated using pharmacological agents. Another HRCT study has been used to directly assess response to therapy as well as survival [30]. More recently, quantitated CT has been explored with some interesting findings. In one such retrospective IPF study, textural CT patterns derived from HRCT were shown to correlate with lung function [31]. In another, a computer algorithm was used to quantify CT changes in IPF patients, and these were shown to be associated with mortality and add further information to the combined physiological index (COI) or GAP scores [32]. Similarly, another group used textural analysis using dual-energy contrast CT in patients with ILD, showing mortality associations [33]. In contrast, our imaging approach is novel and has the potential advantage of exploiting the functional data and sensitivity of PET [34]. Nonetheless, it is recognized that the underlying CT changes may affect the PET CT signal per se, as has been previously recognized [35]. Differences observed in SUV as used in this paper could be due to a combination of differences in tracer uptake and tissue density. However, it is worth noting that the SUV measures presented in this paper can be computed with standard software.

Although HRCT is essential for diagnosis, the PET signal was important for prognosis when measured from regions where HRCT lung parenchyma findings were normal. In a retrospective analysis, it was shown that increased ^18^F-FDG in normal lung was associated with poor survival in ILD patients [36]. This is in keeping with another study that showed that in IPF patients normal lung parenchyma as demonstrated on HRCT showed higher ^18^F-FDG uptake than control patients [37]. This may suggest that PET can detect disease signals before anatomical changes on HRCT are revealed, and as such, PET, in addition to detecting severe disease, may be detecting early or mild disease.

The precise cellular mechanisms underlying the observed FDG-PET signal in IPF are as of yet unknown. We and others have considered the possibility that the FDG signal is generated by inflammatory cells in the alveolar space that may not be seen on lung biopsy, and act as sentinels of lung damage. However, there is accumulating evidence that glucose uptake plays a fundamental role in the fibrotic process. The facilitative glucose transporter 1 GLUT-1 is up-regulated on fibroblasts in the fibrotic regions of both patients with IPF and mice that are subjected to a fibrosis-inducing bleomycin treatment [38]. GLUT1 is induced by transforming growth factor (TGF)-β in fibroblast lines and primary cells and is required for the profibrotic effects of T GF-β. Further evidence that metabolically active cells, taking up increased amounts of glucose, are present in the lungs of IPF patients come from the increased lactic acid levels found in the lungs of IPF patients compared to healthy and COPD controls [39]. This lactic acid may contribute to the pathogenesis of the disease by activating TGF-β and driving collagen production from fibroblasts [39]. Regardless of the underlying mechanism, the utility of FDG-PET as a prognostic marker in IPF remains valid and reflects the presence of metabolically active cells in the lungs of these patients.

There are many technical factors that need to be appreciated in this type of imaging study, such as density-induced artefacts, a varying blood fraction in the lung, and respiratory motion due to free breathing during the acquisition. The methodological considerations involved in tackling these challenges have been dealt with elsewhere [7, 8, 40–42]. Furthermore, respiratory gating would allow reducing the effect of the breathing, albeit at the expense of increased noise. Despite these challenges, we were able to make significant survival observations using routine PET measurements. There are a number of standard clinical ^18^F-FDG measurements that can be acquired. The three measurements we have performed are easily implemented and are used to quantify the glucose uptake in routine clinical practice. The advantage of assessing glucose uptake in this way is that use of a background ^18^F-FDG region allows standardization of measurements. We show that TBR in IPF patients is the most prognostic of our three measurements. Our results are in keeping with the previous findings from a dual time point imaging PET study (imaging at 1 and 3 h post-injection of ^18^F-FDG), where a single SUV measurement at 1 h was not found to have an association with survival. However, with the addition of increased SUV information, (the additonal data from the 2nd time point in the dual time point study, and the ratio of SUVs in our study) it did allow for an association with survival [43]. In another interesting study [44], there was a lack of association between SUV_max_ (and mean) with IPF survival, in keeping with our findings. Only more complex measurements of ^18^F-FDG (e.g., tumor lesion glycolysis and metabolic lesion volume), which are more arbitrary, showed any significant survival associations.

Other study limitations include the fact that the timing of the PET study was not always at the time of diagnosis and thus patients would have varying stages of disease. Our data imply that prognosis may be determined at various stages of disease. What would be of particular interest would be an investigation of the use of serial PET scanning in the disease setting. Similarly, serial measurements in PFT (e.g., FVC) and other indicators (e.g., respiratory, hospitalization, preceding change in physiology, disease trajectory some of which are not validated biomarkers) could be assessed in the future. However, it is recognized that there is inherent variability in PFT and PET measurements that serial imaging cannot entirely correct. Another challenge in the IPF patient population is the difficulty in differentiating the contribution of IPF to death from co-morbidities, further compounded by the regular palliative setting of IPF patients at the end of life. For these reasons, we adopted a recognized approach in IPF, and used the all-cause mortality as also in the original GAP analyses [16]. Finally, minimizing bias is challenging in this type of study. The use of test and validation populations can be helpful, but instead we used a k-fold cross-validation technique, which is appropriate in a population size such as in our study [25]. We further minimized bias using median value as cut-offs.

## Conclusions

We have shown that high pulmonary uptake of ^18^F-FDG (TBR) is associated with mortality in our population of IPF patients. These PET findings were statistically independent of GAP survival associations, but were also shown to act synergistically with the GAP classification to help further risk stratify IPF patients.

## Electronic supplementary material

ESM 1 (PDF 221 kb)
