# Supplementary material for: Pulmonary 18F-FDG uptake helps refine current risk stratification in idiopathic pulmonary fibrosis (IPF)
Source: Eur J Nucl Med Mol Imaging. 2018 Jan 16;45(5):806–15. doi: 10.1007/s00259-017-3917-8 (PMC5978900; doi:10.1007/s00259-017-3917-8)
Supplement: Supplementary file 1 — (PDF 221 kb) [file 259_2017_3917_MOESM1_ESM.pdf]

SUPPLEMENT

SUPPLEMENTAL METHODS

Cross-validation

Cross validation technique entailed random partitioning of the cohort into 4 similar sized sub-groups keeping the proportion of events (alive and death) in each sub-group similar to the entire population. Using a leave-one-out approach, three sub-groups were combined as a "training" set and the one sub-group left out was used as a "validation" set. The median value from the "training" set was identified and its ability to predict prognosis (Kaplan Meier analysis) was noted. It was further used as a cut-off to stratify patients as having a poor- or good-prognosis in the "validation" set. This cross-validation (leave-one-out) process was performed 4 times (the folds), with each of the 4 sub-groups used exactly once as a "validation" set. PET markers that did not show consistency in the magnitude and direction of cut-offs (i.e. poor prognosis patients in each fold that were consistently on the same side of the threshold as the whole cohort) were rejected from further analysis. The poor- and good- prognosis patients defined for each "validation" set (as described above) were combined to give the complete cohort and further assessed using Kaplan Meier survival analysis to determine statistical significance after cross-validation. The advantage of this method is that all observations are used for both training and validation, and each observation is used for validation exactly once.

Correlation between PET and PFT

The correlation between FDG PET (SUVmax, SUVmin, TBR) and PFT (FVC, TLCO) were assessed using Spearman's rank order correlation.

Additional Modelling PET data with GAP analysis- GAP stage "risk-stratified by" PET

Another modelling that was employed in addition to the above modified GAP calculation was GAP stage risk-stratified by PET variable (SUVmax or SUVmin or TBR that demonstrated the best prognostic ability in our study). For each patient, the best PET marker was binarised, based on the median cut-off, as an adverse (coded as 1) or favourable (coded as 0) PET signal, then the original GAP stage was stratified as either "GAP stage P-" if the PET marker was favourable or "GAP stage P+" if the PET marker was adverse.

SUPPLEMENTAL RESULTS

Correlation between PET and PFTs

There were significant negative correlations between the PET markers (e.g. SUVmax and SUVmin) and PFT (e.g. FVC). TBR did not show any significant correlation with PFT (e.g. FVC, TLCO) further highlighting the potential to provide unique, additional, complementary information to known lung physiological parameters.

SUVmax and FVC (rs = -0.342, p<0.001),

SUVmin and FVC (rs = -0.271, p=0.004)

TBR and FVC (rs = -0.051, p=0.591).

SUVmax and TLCO (rs = -0.198, p=0.058),

SUVmin and TLCO (rs = -0.061, p=0.566)

TBR and TLCO (rs = -0.173, p=0.100).

There was a significant association (Supplement Fig 1) between FVC and survival (FVC, p=0.001), where patients with FVC > 72.5% predicted (median) had good prognosis and patients with FVC <= 72.5% predicted had poor prognosis with a hazard ratio of 2.5 (95% CI: 1.4 - 4.5)

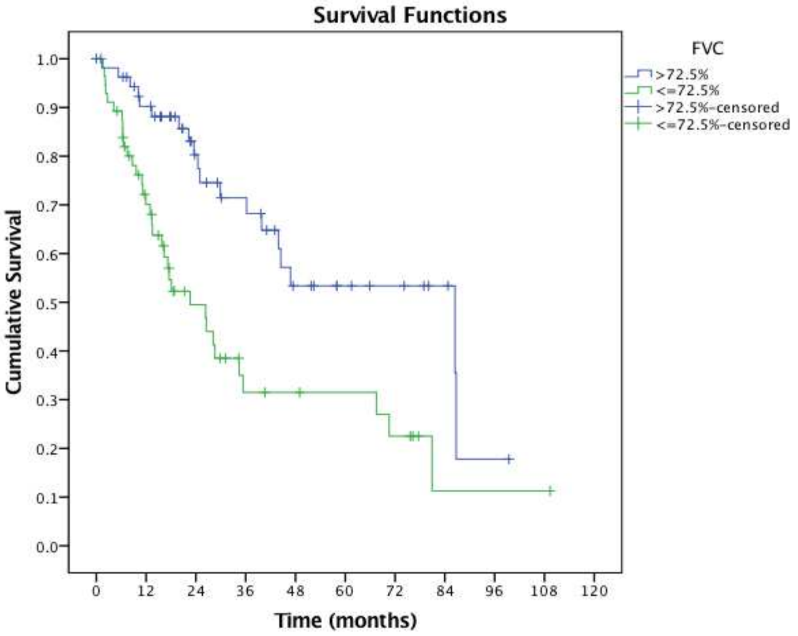

Supplement Fig 1.

FVC (cut off value of 72.5) and survival in IPF patients

In the population of 92 IPF patients who were able to perform measurement of TLCO, there was a significant association (Supplement Fig 2) between TLCO and survival (TLCO, $p<0.001$), where patients with TLCO > 45.0 % predicted (median) had good prognosis and patients with TLCO <= 45.0 % predicted had poor prognosis with a hazard ratio of 3.4 (95% CI: 1.8 - 6.8)

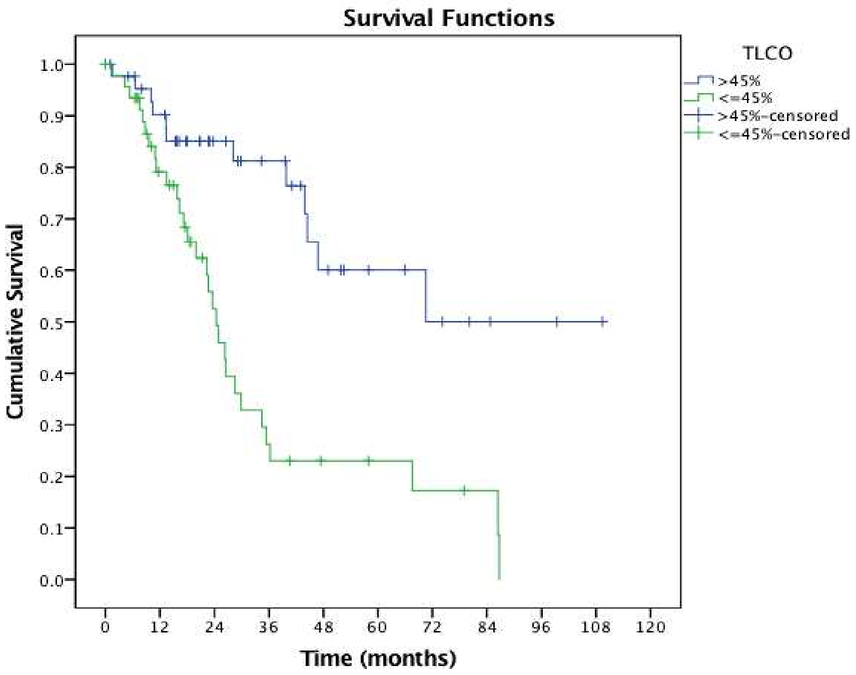

Supplement Fig 2.

TLCO (cut off value of 45 % predicted) and survival in IPF patients

GAP stage risk-stratified by PET.

Supplement Table 1

GAP Stage risk-stratified by PET

GAP I TBR < Median, (*n*=18)	11%
GAP I TBR >= Median, (*n*=17)	46%
GAP II TBR < Median, (*n*=25)	48%
GAP II TBR >= Median, (*n*=25)	72%
GAP III TBR < Median, (*n*=13)	41%
GAP III TBR >= Median, (*n*=14)	89%

**The difference in the survival curves based on the above GAP stage risk stratified by PET approach yielded a p<0.001 (Log-rank test)*

As shown in the supplement table above, GAP stage I is further risk-stratified based on PET (TBR) as “GAP I P-” (GAP I TBR < Median) and “GAP I P+” (GAP I TBR >= Median); The 3 year mortality for the original GAP stage I was 24% (Table 2), whereas the 3 year mortality for “GAP I P-” and “GAP I P+” are 11% and 46% respectively.

The 3 year mortality for the original GAP stage II was 56% (Table 2), whereas the 3 year mortality for “GAP II P-” and “GAP II P+” are 48% and 72% respectively (Table 2). The 3 year mortality for the original GAP stage III was 65% (Table 2), whereas the 3 year mortality for “GAP III P-” and “GAP III P+” are 41% and 89% respectively.
